# Design and Content Validation using Expert Opinions of an Instrument Assessing the Lifestyle of Adults: The ‘PONTE A 100’ Questionnaire

**DOI:** 10.3390/healthcare11142038

**Published:** 2023-07-16

**Authors:** Francisco Javier Pérez-Rivas, Jennifer Jiménez-González, Marianela Bayón Cabeza, Susana Belmonte Cortés, Marta de Diego Díaz-Plaza, Julia Domínguez-Bidagor, David García-García, Juana Gómez Puente, Tomás Gómez-Gascón

**Affiliations:** 1 Grupo de Investigación UCM “Salud Pública-Estilos de Vida, Metodología Enfermera y Cuidados en el Entorno Comunitario”, Departamento de Enfermería, Facultad de Enfermería, Fisioterapia y Podología, Universidad Complutense de Madrid, 28040 Madrid, Spain; tomas.gomez@salud.madrid.org; 2 Red de Investigación en Cronicidad, Atención Primaria y Promoción de la Salud—RICAPPS—(RICORS), Instituto de Salud Carlos III, 28029 Madrid, Spain; 3 Instituto de Investigación Sanitaria Hospital 12 de Octubre (Imas12), 28041 Madrid, Spain; 4 Programa de Doctorado ‘Cuidados en Salud’, Facultad de Enfermería, Fisioterapia y Podología, Universidad Complutense de Madrid, 28040 Madrid, Spain; jennjime@ucm.es (J.J.-G.); daviga27@ucm.es (D.G.-G.); 5 Área de Procesos y Calidad, Gerencia Asistencial de Atención Primaria, Servicio Madrileño de Salud, Consejería de Sanidad, Comunidad de Madrid, 28035 Madrid, Spain; marianela.bayon@salud.madrid.org; 6 Área de Nutrición y Estilos de Vida, Subdirección de Prevención y Promoción de la Salud, Dirección General de Salud Pública, Consejería de Sanidad, Comunidad de Madrid, 28002 Madrid, Spain; susana.belmonte@salud.madrid.org (S.B.C.); marta.diego@salud.madrid.org (M.d.D.D.-P.); 7 Unidad Técnica Promoción de Salud, Subdirección General Prevención y Promoción de Salud, Dirección General de Salud Pública, Comunidad de Madrid, 28002 Madrid, Spain; julia.dominguez@salud.madrid.org; 8 Centro de Salud Eloy Gonzalo, Gerencia Asistencial de Atención Primaria, Servicio Madrileño de Salud, Consejería de Sanidad, 28010 Madrid, Spain; jgomezp@salud.madrid.org; 9 Fundación para la Investigación e Innovación Biosanitaria de Atención Primaria (FIIBAP), 28003 Madrid, Spain; 10 Facultad de Medicina, Universidad Complutense de Madrid, 28040 Madrid, Spain

**Keywords:** promotion of health, health questionnaire, primary healthcare, expert opinion, content validation, Aiken V test, lifestyle, habits, food habits, physical exercise, smoking, alcohol, drugs, emotional wellbeing, safety, accidents

## Abstract

Lifestyle, a major determinant of health status, comprises a number of habits and behaviours that form a part of daily life. People with healthy lifestyles have a better quality of life, suffer less disease, and have a longer life expectancy. This work reports the design and content validation of a questionnaire—the ‘PONTE A 100’ questionnaire—assessing the lifestyle of adults. This collects information across five dimensions—‘Eating Habits’, ‘Physical Activity’, ‘Smoking and use of Alcohol and other Drugs’, ‘Emotional Wellbeing’, and ‘Safety and Non-intentional Injuries’—via the answering of a total 33 items. Psychometric validation of the instrument’s content was obtained via expert opinions. This was performed by two rounds of assessment and involved 34 experts representing different health science disciplines (mean experience, 27.4 ± 9.4 years). At the end of each round, adjustments were made according to their recommendations. Agreement between the experts was examined using the Aiken V test. A final V value of 0.95 (95% CI, 0.90–1.00) was obtained for the questionnaire as a whole, highlighting the validity of its content. The questionnaire would therefore appear to be an appropriate instrument for assessing the lifestyle of adults.

## 1. Introduction

### 1.1. Lifestyle: Definition and Impact on Health

The World Health Organization defines lifestyle as a way of living based on identifiable patterns of behaviour, which are determined by the interplay between an individual’s personal characteristics, social interactions, and socioeconomic and environmental living conditions [1]. Back in the 1970s, Lalonde established lifestyle as the factor with the greatest influence on health, explaining 47% of mortality [2]. Since then, many studies have provided solid evidence highlighting its impact on health in general [3] and on disease burden [4]. The cause–effect relationship between different types of behaviour and health status has been confirmed in studies focusing on risk factors such as smoking [5,6], the use of alcohol [7,8], low physical activity levels and sedentary behaviour [9,10], inadequate diet [11,12], and emotional stress [13,14]. Although these studies all report these risk factors to have an impact on health, indices that take into account their combined influence might better reflect the true risk a person faces [15,16].

### 1.2. Lifestyle and Primary Healthcare

For most people, primary healthcare is the most accessible type of healthcare. It is at this level that concepts of good health and disease prevention need to be driven home [17,18]. Primary healthcare professionals, in particular nurses, should be promoting the healthiest lifestyle possible to the populations they serve. They therefore need appropriate tools for assessing lifestyle, promoting health, and preventing disease.

### 1.3. Lifestyle Questionnaires

A number of questionnaires that investigate lifestyle and the determinants of health are used at the international level [19,20]. Generally, however, these have a more epidemiological focus and aim to help in health policy decision-making; they are not designed with a clinical focus that might be of use to health workers. Questionnaires with a more clinical orientation for use with adult populations do exist [21,22,23,24], and some do indeed assess different dimensions related to lifestyle, but none contemplates all the dimensions that international bodies now understand to have an impact on it [25,26].

### 1.4. Validation by Expert Opinion

All instruments used to obtain data from populations should be carefully designed in terms of the dimensions and components covered. They should also undergo validation before entering use [27]. The ‘validity’ of a questionnaire refers to its capacity to measure that for which it was designed. The validity of its content is the degree to which the items it contains are representative of the theoretical construct that the questionnaire is designed to assess. Content validation—which is crucial in the development of a new questionnaire—is based on the definition of the areas or dimensions covered. A questionnaire can be deemed valid in terms of its content if it contemplates all aspects related to the concept under study [28]. Content validation is usually tackled by seeking expert opinion. Experts in the field are asked to propose/approve the dimensions and items included in a questionnaire, to eliminate those deemed irrelevant and to make any required adjustments to the text of those incorporated [29].

The aim of the present work was to design and content-validate via expert opinion a questionnaire—in Spanish known as the Ponte a 100 (PA100—a play on words roughly meaning ‘Get into top gear!’) questionnaire—for assessing the lifestyle of adults, with a synthetic scale (0–100) for assigning a ‘Lifestyle Index Value’ (LIV).

Future articles will provide information on the analysis of the psychometric properties of the questionnaire (internal consistency, reliability according to the form of administration—self-administered or hetero-administered—, intra- and inter-observer reliability, construct validity…), as well as the evaluation of the usefulness/usability of the questionnaire by clinicians, with the aim of providing all the information on the questionnaire for its application in clinical practice.

## 2. Materials and Methods

### 2.1. Study Stages

**Figure 1:**
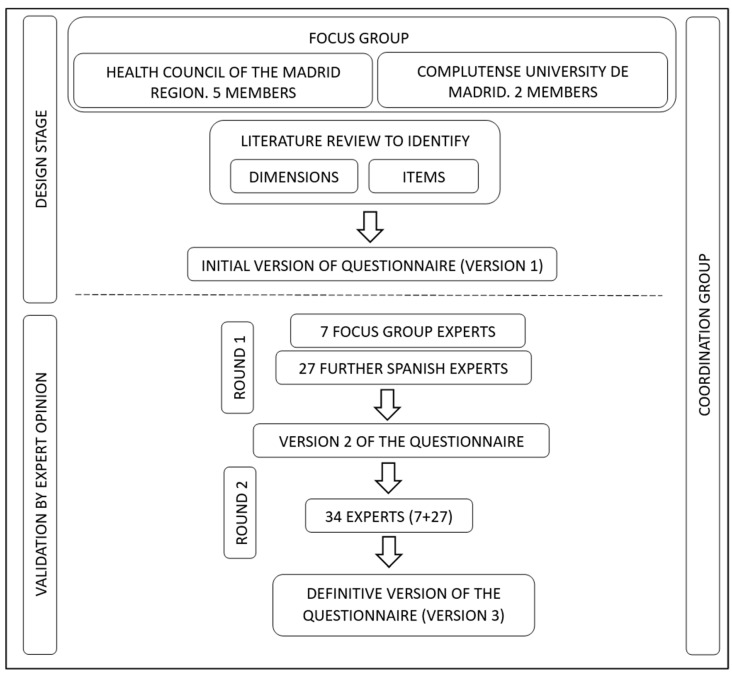
Stages followed in the design and validation of the PA100 questionnaire.

Figure 1 provides a summary of the stages followed in this descriptive, psychometric validation of the content of the PA100 questionnaire. 

#### 2.1.1. Stage 1: Questionnaire Design

The designers of the questionnaire belonged to a focus group composed of three nurses, two psychologists, a physician, and a pharmacist, all of whom were affiliated with either the Consejería de Sanidad de la Comunidad de Madrid (Health Council of the Madrid Region) or the Faculty of Nursing, Physiotherapy and Chiropody at the Complutense University of Madrid. All members had clinical, teaching, research, and/or management experience related to the importance of lifestyle. One Health Council-based and one University-based group member acted as coordinators with assistance from an University-based doctoral student. The substages of the design process are described below.

1. Literature review

The aims of this review were:

- to examine existing questionnaires/tools for the assessment of lifestyle, noting their format, the dimensions covered, and the assessment system used;
- to collate evidence justifying the dimensions and items required and the assignment of LIV points to them, their sum providing an overall numerical value for lifestyle.

Relevant literature was sought in the MEDLINE, CINHAL, LILACS, Dialnet, Scopus, Web of Science, TESEO databases, Cochrane Library, and Joanna Briggs Institute databases, in the Recolecta and Scielo repositories and in the Dynamed and Nursing + Evidence Summaries, using the Trip and Epistemonikos metasearch engines. The Spanish search terms used were the Health Science Descriptor (DeCS in Spanish) terms ‘Estilo de vida’, ‘Encuestas y Cuestionarios’, and ‘Adulto’; the English terms used were the major Mesh terms ‘Lifestyle (or Life Style)’, ‘Questionnaire’, and ‘Adult’.

2. Definition of dimensions and items

Lifestyle is a multidimensional concept. The individual dimensions that would allow a combined view of adult lifestyle were therefore identified first. The items included in each dimension were chosen following the recommendations of Argimon [28] (i.e., avoiding ambiguous questions, not using vague terms, not formulating two questions in one, keeping them short, using simple language, and keeping the number of questions to a minimum). The selection of dimensions and their items was based on clear evidence with respect to their impact on disease burden and quality of life.

3. Initial version of the questionnaire

Once the above stage was complete and following a number of meetings and discussions, the focus group proposed an initial version of the questionnaire (Version 1).

#### 2.1.2. Stage 2. Content Validation

1. Participants

Thirty-four experts were selected based on their experience in and knowledge of the different dimensions in the questionnaire. This included the seven members of the focus group (all from the Madrid Region) and 27 others from the Spanish regions of Andalusia, Aragon, Asturias, the Balearic Islands, the Canary Islands, Catalonia, the Valencia Region, Extremadura, Madrid, and the Basque Country. Sixteen of these experts were nurses (47%), 10 were physicians (29%), 4 were psychologists (12%), and 4 were pharmacists (12%). The expert group’s mean number of years of professional experience was 27.4 ± 9.4. At the time of recruitment, the main duties of the experts included those of university lecturers (n = 9), front line healthcare providers (9), management of primary care (6), public health technicians (4), public health managera (2), and researcher/research managers (4). Some 59% possessed a doctorate in the area of health sciences (Table 1). All experts were sent an e-mail inviting them to take part, a description of the aims of the study, the instructions needed to complete their assessment tasks, and the required evaluation template (see below).

2. Procedure
  2.1. Round 1: Content validation of Version 1

For content validation, a template in Word format was used to record the assessment of the questionnaire items by all 34 experts (performed individually with no inter-expert contact) in terms of the criteria of Escobar and Cuervo [29]:

- sufficiency, i.e., that the collection of items contained in a dimension are sufficient to assess the variable under scrutiny;
- coherence, i.e., that the items have a logical relationship with the dimension being assessed;
- clarity, i.e., that the items are easily understood (adequate syntax and semantics);
- relevance, i.e., that an item is important.

Each item was assigned a value on the 1–4 Likert scale in terms of how well these criteria were met: 1: not met, 2: met at low level, 3: met at an intermediate level, and 4: met at a high level. Fields were also provided to record qualitative valuations for each item and for answering two generic questions:

- at the end of each dimension: would you add any other item? (no/yes, specify);
- at the end of the questionnaire: would you add any other dimension (no/yes, specify).
  2.2. Round 2: Reaching expert consensus

At this point, the experts were made privy to the statistical analysis of the first round results and the recommendations made by their peers (presented anonymously). According to the results obtained and observations made in Round 1, the group coordinators made changes to the questionnaire, resulting in Version 2. This version included the new dimension ‘Safety and Non-intentional Injuries’ and grouped the dimensions ‘Alcohol use’ and ‘Smoking’ into the single dimension ‘Smoking and use of Alcohol and other Drugs’, along with other more minor changes.

The experts were sent a new template for assessing Version 2, including the items in its new dimension. This was performed as described above, although the dimensions and items for which consensus had already been reached in Round 1 were not re-assessed. Only these two rounds of assessment were required for major consensus to be reached. During the experts’ assessment of Version 2, they made only minor qualitative changes to a few item texts. This resulted in the definitive Version 3.

### 2.2. Statistical Analysis

Quantitative variables (scores given by the experts for each criterion, dimension, and item) were recorded as means ± standard deviation; qualitative variables were recorded as frequencies and percentages. The Aiken V test [30] was used to calculate the agreement between experts, thus quantifying the validly of the questionnaire’s content; the resulting values range from 0 (no agreement) to 1 (full agreement between all experts). Values close to 1 indicate validity. The 95% confidence intervals were calculated for V according to Penfield and Giacobbi [31], using an ad hoc program in Microsoft Excel 365^®^ (Microsoft, Redmond, WA, USA). Validity was accepted when the lower end of the interval was >0.7 [31,32].

## 3. Results

### 3.1. Questionnaire Design

The conceptual and theoretical design stage involving the literature review and meetings of the focus group led to the proposal of Version 1 of the questionnaire. This had five dimensions—‘Food habits’, ‘Physical activity’, ‘Smoking’, ‘Alcohol consumption’, and ‘Emotional wellbeing’—encompassing a total of 22 items (Table 2). All the dimensions had the same weighting, i.e., 20 LIV points (the sum of the points assigned to the items in a dimension). The sum of the LIV points for all items would therefore provide a final LIV with a maximum value of 100.

### 3.2. Content Validation

#### 3.2.1. Modifications Made to the Questionnaire after Round 1 of Expert Assessment

Table 3 shows the descriptive analysis of the scores awarded by the experts in Round 1 for all dimensions (this table also includes the dimension ‘Safety and Non-intentional Injuries’, which was added after Round 1 given the results obtained in that round, and assessed in Round 2 [see below]). The qualitative analysis made by the experts suggested the following amendments be made to the questionnaire, thus helping to design Version 2.

The dimensions ‘Smoking’ and ‘Alcohol Use’ were grouped into a new dimension ‘Smoking and use of Alcohol and other Drugs’ (note that this included a new item ‘Use of other drugs’). The LIV points for this dimension were also modified to allow negative points to be obtained given the impact of toxic habits on health (−5, −10, or −15 depending on consumption). 

The new dimension ‘Safety and Non-intentional Injuries’ was also added. This was included to take into account important health aspects such as compliance with road safety recommendations or of water-use regulations designed to avoid drowning and other accidents, etc. These are of particular importance with respect to young people. 

The LIV points for all dimensions were modified according to the recommendations of some of the experts and in light of the data for Spain in the document ‘Global burden of 87 risk factors in 204 countries and territories, 1990–2019: a systematic analysis for the Global Burden of Disease Study 2019′ [33]. 

Following the recommendations made by some experts, the text of some items was changed to improve understanding. 

Thirty-two experts (91.42%) believed it necessary to include the new dimension ‘Safety and Non-intentional Injuries’ in the final version and to provide the possibility of scoring negative LIV points for the items in the dimension ‘Smoking and use of Alcohol and other Drugs’. A total of 94.3% considered it pertinent to include the item “Use of other drugs” in the latter dimension. 

Table 4 shows all the modifications made. Table 5 shows the percentage agreement between the experts for each of the modifications made. 

Following this round of assessment, the resulting version—Version 2—also had five dimensions, with a total number of LIV points of 100.

#### 3.2.2. Modifications Made to the Questionnaire after Round 2 of Expert Assessment and Production of Version 3

The new items and dimension in Version 2 of the questionnaire were re-assessed as above, although those for which consensus had been reached in Round 1 were not re-assessed (see the corresponding section in Table 3). Minor qualitative changes were also made. This led to the production of the definitive Version 3 of the questionnaire.

The final mean score for the dimensions was 3.86 ± 0.34, with practically no differences between the dimensions. Among the analysed criteria, item coherence obtained the highest score (mean 3.94 ± 0.05) followed by item relevance (3.91 ± 0.08). Dimension sufficiency and item clarity also returned high scores (both means > 3.5). Two scores of <3.5 were recorded: sufficiency for the dimension ‘Food Habits’ (3.44 ± 0.82) and clarity for the item ‘red meat’ in that dimension (3.42 ± 0.81).

Table 6 records a final Aiken V score of 0.95 (95% CI: 0.90–1.00) for the questionnaire. All the dimensions returned a V of >0.95, except for ‘Safety and Non-intentional Lesions’, which had a V of 0.87 (95% CI: 0.80–0.94). All V values were >0.7 (greater than the lower limit of the 95% CI). Among the criteria, item coherence and item relevance enjoyed the greatest agreement. 

Supplementary File S1 contains the final questionnaire in Spanish; Supplementary File S2 contains the English translation.

The definitive version of the questionnaire (Version 3) thus comprised five dimensions: ‘Food habits’, ‘Physical Activity’, ‘Smoking and use of Alcohol and other Drugs’, ‘Emotional Wellbeing’, and ‘Safety and Non-intentional Injuries’ (Table 7). The maximum LIV score possible was 100 (providing the Spanish name for the questionnaire: Ponte a 100).

The final dimension of ‘Food Habits’ was formed by 11 items that collect information on the consumption of the main food groups with evidence-based positive effects on health (vegetables, fruit, wholemeal cereals, virgin olive oil, pulses, fish/seafood, and water) as well as those that pose health risks (red and processed meats, processed/ultra-processed foods, and sugary/sweetened drinks). Each item is assigned LIV points depending on its health impact.

LIV points for the final dimension of ‘Physical Activity’ were awarded according to current recommendations, i.e., in terms of whether physical activity is intense, moderate, or a combination of both (as shown by the complementary nature of items 1 and 2); whether it involves exercise for increasing muscular strength; and whether periods of walking/stretching etc. are performed every 60–90 min during times of sedentary behaviour. 

The final dimension of ‘Smoking and use of Alcohol and other Drugs’ was altered to contemplate negative LIV points for risk-associated behaviours.

The final dimension of ‘Emotional Wellbeing’ assesses self-acceptance/self-concept, satisfaction/personal development, emotional balance/control of stress, motivation/attitude, the capacity to face problems, interpersonal relationships/family structure/social support, affective needs, sleep/rest, and leisure/entertainment.

The final dimension of ‘Safety and Non-intentional Injuries’ assesses features related to risk-associated behaviour, e.g., respect for traffic norms as a driver, passenger, and pedestrian; driving under the influence of toxic substances; respect for water activity regulations; and following basic recommendations to prevent domestic accidents.

## 4. Discussion

The use of expert opinions is a common way to validate the content of questionnaires; it provides a way to determine how well an instrument measures variables of interest [29]. Recommendations vary regarding the number of experts that should form a content validation panel, the influencing factors including the panel members’ level of experience, their diversity of knowledge, the characteristics of the instrument under observation, and the goals set [34]. Generally, a panel of 25–30 experts is regarded as sufficiently large [35]. In the present work, the panel consisted of 34 experts, all selected on the basis of their experience and knowledge. 

The methodology used to determine the validity of the questionnaire, i.e., including the checking of dimension sufficiency and item coherence, clarity, and relevance [29], has been used on many occasions in other studies in the field of health science [36,37,38,39,40] and indeed in other contexts [34,41,42]. The Aiken V test is also reported to be the best method for determining content validity [43]. It has also been used in many studies validating other instruments [38,39,40,44,45,46,47,48,49].

The present results indicate the content of the PA100 questionnaire to be valid. The final agreement between the experts was very strong, with V values of >0.8 consistently recorded for dimension sufficiency and item coherence, clarity, and relevance. The overall final V value of 0.95 (95% CI 0.90–1.00) confirms the questionnaire to be valid for assessing lifestyle. This value is higher than that reported by other authors in their validation of other instruments (Narayan [44], V = 0.65; Navarro [45], V = 0.78; Caballero [46], V = 0.83). These latter authors encountered more difficulty in determining which items and/or descriptors allowed for the adequate measurement of the object of interest. However, in other studies, high V values have been reported (Curcio [47], V = 0.92; Farhat [48], V = 0.95; Mamani [40], V = 0.96; and Feligreras [49], V = 1).

Finally, the present work has some limitations. The experts in this study were all Spanish; the use of the questionnaire in countries with different types of health behaviours or needs might pose some problems. Nonetheless, all their recommendations were evidence-based, suggesting the questionnaire could be used elsewhere. It is also possible that the answers provided by the experts could have been subjectively conditioned by their background (academic, front line health worker, researcher, or manager). However, the aims of the work and of the final questionnaire were made very clear, helping to focus any assessments they made.

## 5. Conclusions

The PA100 questionnaire would appear to be a valid and adequate instrument for assessing the lifestyle of adults, allowing habits that can be improved to be identified. The LIV provides a numerical value on a scale of 0–100 for an individual’s lifestyle, with 100 representing ‘completely healthy’. This should allow the impact of lifestyle modifications to be monitored and made clear to patients/healthcare clients. In the primary healthcare setting, this easy-to-use tool could be of great interest to nurses and other health professionals, allowing them to better promote healthy lifestyles among those they serve.

## Figures and Tables

**Table 1:** Characteristics of the experts forming the validation panel.

Expert	Academic Training	Academic Degree	Work Position	Years of Professional Experience
1	Nurse	Doctorate	University lecturer	31
2	Physiologist	Bachelor	Public health technician (PHT)	28
3	Nurse	Bachelor (FCN)	Front line health worker	31
4	Psychologist	Bachelor	Public health technician (PHT)	16
5	Nurse	Master	Primary care manager	23
6	Physician	Doctorate	Research manager	38
7	Pharmacist	Doctorate	Public health manager	34
8	Nurse.	Doctorate	University lecturer	32
9	Nurse	Bachelor (FCN)	Front line health worker	32
10	Physician	Doctorate	Front line health worker	50
11	Nurse	Master/PhD student	Primary care manager	20
12	Physician	Doctorate	Researcher and University lecturer	40
13	Nurse	FCN	Front line health worker	30
14	Physician	Doctorate	University lecturer	25
15	Nurse	FCN	Front line health worker	31
16	Physician	Doctorate	Front line health worker University lecturer	25
17	Physician	Doctorate	University lecturer	40
18	Nurse	Doctorate	Researcher	14
19	Pharmacist	Doctorate	University lecturer	30
20	Nurse	FCN	Front line health worker	34
21	Psychologist	Doctorate	Researcher	19
22	Pharmacist	Doctorate	University lecturer	25
23	Nurse	Doctorate	Front line health worker and university lecturer	36
24	Nurse	Master/PhD student	Primary care manager	10
25	Psychologist	Doctorate	Primary care manager	28
26	Physician	Bachelor (FCM)	Public health technician (PHT)	16
27	Nurse	Bachelor (FCN)	Front line health worker	28
28	Nurse	Doctorate	Primary care manager	29
29	Physician	Doctorate	Front line health worker and university lecturer	38
30	Nurse	Doctorate	Primary care manager	19
31	Physician	Bachelor (FCM)	Public health manager	19
32	Physician	Bachelor (PMPH)	Public health technician (PHT)	15
33	Pharmacist	Doctorate	University lecturer	10
34	Nurse	Doctorate	University lecturer	36

FCN: specialist in family and community nursing, FCM: specialist in family and community medicine, PMPH: specialist in preventive medicine and public health, PHT: specialist in health promotion and disease prevention at community level.

**Table 2:** Structure of the PA100 questionnaire: Version 1.

Dimension	Items (No)	LIV Points
Food habits	10	0–20 points
Physical activity	3	0–20 points
Alcohol consumption	1	0–20 points
Smoking	1	0–20 points
Emotional wellbeing	7	0–20 points
Total	22	100 points

LIV = Lifestyle index value.

**Table 3:** Content validity as determined by the experts in the corresponding rounds of assessment (see text): mean (and standard deviation) scores for dimension sufficiency and item coherence, relevance, and clarity.

Dimension	Dimension Sufficiency Mean (Sd)	Item	Item Coherence Mean (Sd)	Item Relevance Mean (Sd)	Item Clarity Mean (Sd)	Total Por Dimension
Food habits	3.44 (0.82)	Vegetables	3.96 (0.20)	3.96 (0.20)	3.73 (0.45)	3.84 (0.33)
		Fruits	4.00 (0.00)	4.00 (0.00)	3.81 (0.40)	
		Wholegrain cereals	3.85 (0.37)	3.85 (0.37)	3.62 (0.50)	
		Processed meats	3.92 (0.27)	3.88 (0.43)	3.58 (0.64)	
		Red meats	3.88 (0.43)	3.96 (0.20)	3.42 (0.81)	
		Fizzy drinks	3.92 (0.27)	3.96 (0.20)	3.58 (0.58)	
		Pulses	4.00 (0.00)	3.92 (0.27)	3.88 (0.43)	
		Fish	3.92 (0.27)	4.00 (0.00)	3.81 (0.40)	
		Nuts	4.00 (0.00)	3.96 (0.20)	3.85 (0.37)	
		Oil	3.92 (0.27)	3.88 (0.43)	3.65 (0.56)	
Physical activity	3.67 (0.48)	Intense physical activity	3.96 (0.20)	4.00 (0.00)	3.73 (0.45)	3.85 (0.32)
		Moderate physical activity	3.96 (0.20)	4.00 (0.00)	3.77 (0.43)	
		Sedentary behaviour	3.85 (0.37)	3.85 (0.37)	3.73 (0.53)	
Alcohol consumption	3.81 (0.40)	Alcohol consumption	4.00 (0.00)	4.00 (0.00)	3.73 (0.53)	3.85 (0.27)
Smoking	3.54 (0.58)	Smoking	4.00 (0.00)	4.00 (0.00)	3.69 (0.58)	
Emotional wellbeing	3.68 (0.48)	Self-concept	3.92 (0.27)	3.83 (0.48)	3.83 (0.64)	3.88(0.36)
		Satisfaction	4.00 (0.00)	3.92 (0.27)	3.88 (0.34)	
		Happiness	4.00 (0.00)	3.83 (0.56)	3.96 (0.20)	
		Emotional wellbeing	4.00 (0.00)	3.88 (0.43)	3.92 (0.27)	
		Motivation	3.88 (0.43)	3.75 (0.61)	3.75 (0.68)	
		Capacity to face problems	3.87 (0.46)	3.83 (0.49)	3.57 (0.79)	
		Interpersonal relationships	3.96 (0.20)	3.96 (0.21)	3.96 (0.20)	
		Affective needs	4.00 (0.00)	3.83 (0.48)	4.00 (0.00)	
		Sleep/rest	3.92 (0.27)	3.88 (0.43)	3.83 (0.38)	
		Leisure/Entertainment	3.81 (0.40)	3.90 (0.30)	3.82 (0.39)	
Round 2 Safety And non-intentional injuries	3.67 (0.56)	Road safety: seatbelt use	4.00 (0.00)	4.00 (0.00)	3.93 (0.25)	3.87 (0.36)
		Road safety: driving and drinking	3.97 (0.18)	3.97 (0.18)	3.87 (0.43)	
		Road safety: adherence to traffic regulations	3.90 (0.31)	3.97 (0.18)	3.83 (0.46)	
		Compliance with water-use regulations	3.90 (0.40)	3.73 (0.64)	3.77 (0.57)	
		Safety at home	3.93 (0.37)	3.83 (0.46)	3.60 (0.81)	
TOTAL	3.63 (0.12)		3.94 (0.05)	3.91 (0.08)	3.77 (0.13)	3.86 (0.34)

**Table 4:** Modifications made to the items and dimensions after Round 1 of expert analysis.

**Food Habits**
The items were modified to appear in terms of frequency of recommended consumption (daily/several times per week/occasionally)
LIV points were adjusted for the items wholegrain cereals, red and processed meat, processed/ultra-processed foods given their weight in the Global Burden of Disease document [33]
Household measurements were added to complement ‘grams per serving’
Extra examples were added for some dimensions to make the text clearer
The consumption of wholegrain cereals was specified as servings/day instead of servings/week
Inclusion of toasted nuts (3 times/seek) with an LIV point value lower than that for the consumption of raw nuts
Red and processed meats were grouped into a single item
It was clarified that refined/sugar-added breakfast cereals are processed foods
A final clarification was made to highlight that processed foods include pre-cooked/ready cooked foods with excess salt, sugar refined oils, or additives/preservatives, but not little-processed foods such as tinned fruit, pickled fruits/vegetables, or jars of pulses
The term ‘sugary/fizzy drink’ was substituted with ‘sugary/sweetened drink’ and assigned an LIV point value depending on rate of consumption
An item on water consumption was included
**Physical Activity**
More inclusive language was used
A new item covering muscular strength training (maximum 4 LIV points) was added with corresponding clarifications
A maximum of 12 LIV points was assigned to aerobic exercise (instead of the original 16)
The example of walking 7000/8000 steps per day was included as an example of moderate activity
The wording of the question on sedentary behaviour was modified
A clarification was made regarding the meeting of physical activity recommendations by adding short periods of activity (of at least 10 min duration) together
**Smoking And Use of Alcohol And Other Drugs**
Smoking was clarified as tobacco use via the consumption of cigarettes, cigars, pipes, e-cigarettes, vaping devices, and hookahs
Smokers were asked about their consumption rate
LIV points ranging from −15 to +15 were contemplated for the degree of consumption
The LIV points for alcohol consumption were set from −10 to +10.
A new item was included to assess the use of ‘other drugs’; their consumption could remove up to 10 points for this dimension
**Emotional Wellbeing**
Motivation was clarified as referring to the will to undertake activities
The item relating to sleep/rest was written from a positive perspective
**Safety And Non-Intentional Injuries**
This dimension, with its 5 items (three relating to road safety and traffic accidents, one to drowning and other water accidents, and one relating to accidents in the home), was added

**Table 5:** Level of agreement between the experts regarding the modifications/inclusions made to Version 1 of the questionnaire.

Dimension	Item	Was the Modification Adequate		Was This New Item Relevant	
		Yes	No	Yes	No
Food habits	Vegetables	91.42%	2.8%		
	Fruits	88.57%	5.7%		
	Wholegrain cereals	88.57%	5.7%		
	Nuts	77.14%	17%		
	Oil	85.71%	5.7%		
	Pulses	91.42%	-		
	Fish/seafood	91.42%	2.8%		
	Red meats	85.71%	8.5%		
	Ultra-processed foods	85.71%	5.7%		
	Sugary/sweetened drinks	85.71%	5.7%		
	Water			82.85%	5.7%
Physical activity	Intense	85.71%	2.8%		
	Moderate	91.42%	2.8%		
	Muscle strengthening exercise			85.71%	5.7%
	Sedentary behaviour	85.71%	8.5%		
Smoking and use of alcohol and other drugs	Use of tobacco	82.85%	11.42%		
	Alcohol consumption	82.85%	8.5%		
	Use of other drugs			94.28%	5.7%
Emotional wellbeing	Self-acceptance/self-concept	91.42%	2.8%		
	Satisfaction/personal development				
	Happiness				
	Emotional balance/stress				
	Motivation/attitude	85.71%	8.5%		
	Capacity to face problems				
	Interpersonal relationships	94.28%	-		
	Affective needs				
	Sleep/rest	88.57%	5.7%		
	Leisure/entertainment				

**Table 6:** Content validity as determined by the experts in the different rounds of assessment (see text): Aiken V values (and 95% CI) for dimension sufficiency and item coherence, relevance, and clarity.

Dimension	Dimension Sufficiency Aiken V (95%Ci)	Item	Item Coherence Aiken V (95% Ci)	Item Relevance Aiken V (95% Ci)	Item Clarity Aiken V (95% Ci)	Total for Each Dimension Aiken V (95% Ci)
Food habits	0.81 (0.73–0.90)	Vegetables	0.99 (0.95–1.00)	0.99 (0.95–1.00)	0.91 (0.85–0.98)	0.95 (0.89–1.00)
		Fruits	1 (0.98–1.00)	1 (0.98–1.00)	0.94 (0.88–1.00)	
		Wholegrain cereals	0.95 (0.90–1.00)	0.95 (0.90–1.00)	0.87 (0.80–0.95)	
		Processed meats	0.97 (0.93–1.00)	0.96 (0.91–1.00)	0.86 (0.78–0.94)	
		Red meats	0.96 (0.91–1.00)	0.99 (0.95–1.00)	0.81 (0.72–0.90)	
		Fizzy drinks	0.97 (0.93–1.00)	0.99 (0.95–1.00)	0.86 (0.78–0.94)	
		Pulses	1 (0.98–1.00)	0.97 (0.93–1.00)	0.96 (0.91–1.00)	
		Fish	0.97 (0.93–1.00)	1 (0.98–1.00)	0.94 (0.88–1.00)	
		Nuts	1 (0.98–1.00)	0.99 (0.95–1.00)	0.95 (0.90–1.00)	
		Oil	0.97 (0.93–1.00)	0.96 (0.91–1.00)	0.88 (0.81–0.96)	
Physical activity	0.89 (0.82–0.96)	Intense physical activity	0.99 (0.95–1.00)	1 (0.98–1.00)	0.91 (0.85–0.98)	0.95 (0.90–1.00)
		Moderate physical activity	0.99 (0.95–1.00)	1 (0.98–1.00)	0.92 (0.86–0.99)	
		Sedentary behaviour	0.95 (0.90–1.00)	0.95 (0.90–1.00)	0.91 (0.85–0.98)	
Alcohol consumption	0.94 (0.88–1.00)	Alcohol consumption	1 (0.98–1.00)	1 (0.98–1.00)	0.91 (0.85–0.98)	0.95 (0.90–1.00)
Smoking	0.85 (0.77–0.93)	Smoking	1 (0.98–1.00)	1 (0.98–1.00)	0.90 (0.83–0.97)	
Emotional wellbeing	0.89 (0.83–0.97)	Self-concept	0.97 (0.93–1.00)	0.94 (0.88–1.00)	0.94 (0.88–1.00)	0.96 (0.91–1.00)
		Satisfaction	1 (0.98–1.00)	0.97 (0.93–1.00)	0.96 (0.91–1.00)	
		Happiness	1 (0.98–1.00)	0.94 (0.88–1.00)	0.99 (0.95–1.00)	
		Emotional wellbeing	1 (0.98–1.00)	0.96 (0.91–1.00)	0.97 (0.93–1.00)	
		Motivation	0.96 (0.91–1.00)	0.92 (0.86–0.99)	0.92 (0.86–0.99)	
		Capacity to face problems	0.96 (0.91–1.00)	0.94 (0.88–1.00)	0.86 (0.78–0.94)	
		Interpersonal relationships	0.99 (0.95–1.00)	0.99 (0.95–1.00)	0.99 (0.95–1.00)	
		Affective needs	1 (0.98–1.00)	0.94 (0.88–1.00)	1 (0.98–1.00)	
		Sleep/rest	0.97 (0.93–1.00)	0.96 (0.91–1.00)	0.94 (0.88–1.00)	
		Leisure/entertainment	0.94 (0.88–1.00)	0.97 (0.93–1.00)	0.94 (0.88–1.00)	
Round 2 Safety and non-Intentional injuries	0.89 (0.83–0.95)	Road safety: seatbelt use	1 (0.98–1.00)	1 (0.98–1.00)	0.98 (0.95–1.00)	0.87 (0.80–0.94)
		Road safety: driving and drinking	0.99 (0.95–1.00)	0.99 (0.95–1.00)	0.96 (0.91–1.00)	
		Road safety: adherence to traffic regulations	0.97 (0.93–1.00)	0.99 (0.95–1.00)	0.94 (0.88–1.00)	
		Compliance with water-use regulations	0.97 (0.93–1.00)	0.91 (0.85–0.98)	0.92 (0.86–0.99)	
		Safety at home	0.98 (0.95–1.00)	0.94 (0.88–1.00)	0.87 (0.80–0.94)	
Total	0.88 (0.81–0.96)		0.98 (0.94–1.00)	0.97 (0.93–1.00)	0.92 (0.86–0.99)	0.95 (0.90–1.00)

**Table 7:** Structure of the definitive version (Version 3) of the questionnaire.

Dimension	Items (No)	LIV Points
Food habits	11	0–25 points
Physical activity	4	0–20 points
Smoking and use of alcohol and other drugs	3	(−35)–25 points
Emotional wellbeing	10	0–20 points
Safety and non-intentional injuries	5	0–20 points
Total	33	100 points

LIV = Lifestyle index value.

## Data Availability

The data presented in this study are available on request from the corresponding author.

## Supplementary Materials

The following supporting information can be downloaded at: https://www.mdpi.com/article/10.3390/healthcare11142038/s1. Supplementary File S1_Questionnaire in Spanish; Supplementary File S2_Questionnaire in English.
