# Supplementary material for: Design and Content Validation using Expert Opinions of an Instrument Assessing the Lifestyle of Adults: The ‘PONTE A 100’ Questionnaire
Source: Healthcare (Basel). 2023 Jul 16;11(14):2038. doi: 10.3390/healthcare11142038 (PMC10379326; doi:10.3390/healthcare11142038)
Supplement: Supplementary file 1 [file healthcare-11-02038-s001.zip › Supplementary File S2_Questionnaire in English.pdf]

Comunidad de Madrid | CONSEJERÍA DE SANIDAD

The *‘PONTE A 100’* healthy lifestyle questionnaire

Healthy lifestyles are those that include habits, behaviours and daily customs that help people remain in adequate physical and mental condition throughout their lifetime. They improve personal wellbeing, help people stay healthy, improve one's capacities, and reduce pain.

DO YOU HAVE A HEALTHY LIFESTYLE?

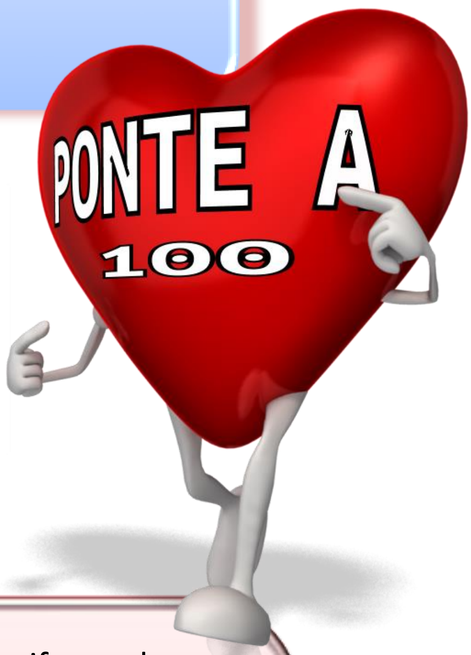

- ✓ **Complete** the following questionnaire to know if you have a healthy lifestyle.
- ✓ The questionnaire's **33 questions are grouped into five sections:** **‘**Food Habits’, ‘Physical Activity’, ‘Smoking and use of Alcohol, and other Drugs, ‘Emotional Wellbeing’ and ‘Safety and Non-intentional Injuries’.
- ✓ **Think each question over and assign yourself points** following the option that best describes your situation over the last **3 months.** There are no right or wrong answers.
- ✓ **Work out your score for each section, as well as your final score** (maximum 100 points).

1.- FOOD HABITS

	Questions	Answers	LIV POINTS
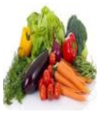	**1. How many servings of vegetables and greens (excluding potatoes) do you eat per day (1 serving* is 150-200g)**<br>* E.g., a large plate of mixed salad, cooked vegetables, vegetable purée or gazpacho, 1 large tomato or 2 carrots. A side dish would be 1/2 a serving.	More than 1 = 2 points<br>1 = 1 point<br>None = 0 points	
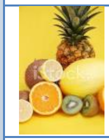	**2. How many servings of fruit do you eat per day?** (1 serving* is 150-200g)<br>* E.g., 1 medium sized orange, apple or banana, 2 small plums, mandarins or apricots, or a bowl of cherries, strawberries, grapes, or a slice of melon, water melon or pineapple.	More than 2 = 2 points<br>1-2 pieces = 1 point<br>None = 0 points	
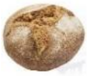	**3. How many of servings* of wholegrain cereals (bread, pasta, rice…) do you have per day?**<br>*E.g., a serving of whole grain bread would weigh 40-0 g (say 2-3 slices);<br>1 serving of wholegrain rice would weigh some 60-80 g (3 tablespoons raw or half a plateful when cooked).)	More than 2 = 3 points<br>2 = 2 points<br>1 = 1 point<br>None = 0 points	
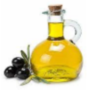	**4. Do you consume at least 3 soup spoonfuls (30 ml) of oil per day (raw or fried)?**	- Yes, of virgin or extra virgin olive = 2 points<br>- Yes, of regular olive oil = 1 point<br>- No, but I consume other types of oil, margarine or butter = 0 points	
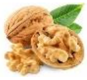	**5. How many servings* of nuts (hazelnuts, almonds, cashews, pistachios) do you have per week?**<br>*1 serving: 1 handful de 20-30 g	Raw: 4 -7 servings/week = 2 points<br>Raw or toasted (1-3 servings) = 1 point<br>I don't eat those quantities or I eat them fried, salted or sweet = 0 points	
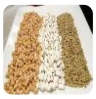	**6. How many servings* of pulses (chickpeas, lentils, beans etc.) do you consume per week?**<br>*1 serving is about 60-80 g raw (1 handful or cupful), 150-200 g cooked (1 medium sized soup plate or 2-3 ladlefuls when served with a sauce)	More than 2 = 2 points<br>1-2 = 1 point<br>None = 0 points	
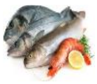	**7. How many servings* of fish/seafood do you have per week?**<br>*1 serving = 100-150 g (1 fillet or medium slice of fish, 3-4 pieces of seafood)	More than 2 = 2 points<br>1-2 = 1 point<br>None = 0 points	
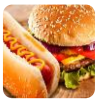	**8. How many servings of red meats* (beef, lamb, pork etc.) or processed meats** (hamburgers, sausages, salami-type sausage, chicken nuggets, etc.) do you have per week?**<br>*1 serving is 100-150 g (a medium sized fillet)<br>**1 serving is 3-4 slices of salami-type sauage,1 hamburger, or 3 meatballs	Less than 3 = 3 points<br>3-4 = 1 point<br>More than 4 = 0 points	
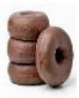<br>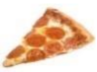	**9. Do you eat any highly- or ultra-processed* foods during the week?**<br>*Sweet or savoury snacks, sweets, commercial sliced bread, sponge cake, buns, cakes, biscuits, sugary/refined breakfast cereals, fish fingers, commercial sauces, pizza, milkshakes, ice cream, instant soups, bouillon cubes, and other precooked or ready cooked foods that contain too much salt, fat, sugar, refined oil, or preservatives and additives. Note that little-processed foods such as tinned fruits, picked vegetables or cooked pulses in jars, etc. are **not** included in this list.	Less than 3 = 3 points<br>3-4 = 1 point<br>More than 4 = 0 points	
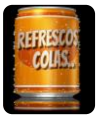	**10. Do you drink sugary/sweetened drinks (including sodas, energy drinks, milkshakes, and bottled/boxed fruit juice every day, or nearly every day?**	No = 2 points<br>Yes, sweetened drinks ('light', 'zero', 'sugar-free') = 1 point<br>Yes, sugary drinks = 0 points	
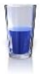	**11. Do you drink water during the day or perhaps with your food?**	Yes, 4 or more glasses*= 2 points<br>Yes, fewer than 4 glasses*= 1 point.<br>No= 0 points<br>* 1 glass = 200 ml	

Total score (Maximum 25 points)

2. PHYSICAL ACTIVITY

	Questions	Answers	LIV POINTS
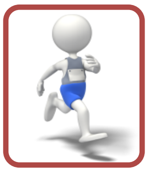	**1.** How many times per week do you do at least 25 minutes of **intense physical activity**?	3 or more = 12 points<br>1-2 = 6 points<br>Never = 0 points	1
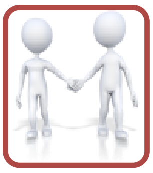	**2.** How many times per week do you do at least 30 minutes **of moderate physical activity**?	5 or more = 12 points<br>3-4 = 6 points<br>1-2 = 3 points<br>Never = 0 points	2
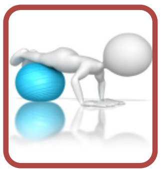	**3.** How many times per week do you do muscle strengthening exercise?*<br>*using weights/dumbbells, exercise machines, resistance bands, or your own body weight (sit-ups on the floor or against a wall, squats, etc.)	2 or more = 4 points<br>1 = 2 points<br>Never = 0 points	3
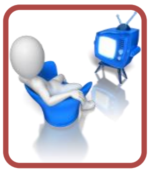	**4.** When you are sat down or laying down during the day do you undertake sessions of active movement/stretching, etc., every **60-90 minutes**?	Always/nearly always: 4 points<br>Sometimes: 2 points<br>Never/hardly ever: 0 points	4

The physical activity times mentioned above can be reached by adding smaller periods of activity (of at least 10 minutes) together.

NOTE: QUESTIONS 1 AND 2 ARE COMPLEMENTARY, ALLOWING FOR COMBINATIONS OF DIFFERENT ACTIVITY TO BE CONTEMPLATED, BUT ALWAYS FOR A MAXIMUM OF 12 POINTS

questions 1+ 2: (**maximum 12 points**) + question 3: (**maximum 4 points**) + question 4: (**maximum 4 points**) = **PHYSICAL ACTIVITY SCORE (Maximum 20 points)**

"Intensity" refers to the effort put into a physical activity.

The experience of intensity depends on how fit a person is. The following are just examples; the experience of intensity may not be the same for everyone.

1.- INTENSE or VIGOROUS. This requires much effort and provokes rapid breathing and a substantially increased heart rate, e.g., jogging, quickly ascending a slope, rapid cycling, aerobics, rapid swimming, and competitive sport such as football, volleyball, basketball, skating or squash.

2.- MODERATE. This requires an increase in breathing without running out of breath, and a perceptible increase in the heart rate, e.g., walking quickly (7000-8000 steps/day), dancing, active participation in sport with children, cycling, swimming for fun.

3. SMOKING AND USE OF ALCOHOL AND OTHER DRUGS

3.1. CONSUMPTION OF ALCOHOL

1. How many **units** of alcoholic drinks do you consume per **week?**

Nº units

ALCOHOL UNITS IN DRINKS

- 1 glass of red, white or sparkling wine (100 ml) = **1** unit
- One small beer or glass of cider (200 ml) = **1** unit
- 1 coffee with liqueur (25 ml) or 1 vermouth (50 ml) = **1** unit
- 1 glass of anise liqueur, sloe gin, or similar (30 ml): **1** unit
- A bottle of beer (330 ml): **1.5** units
- 1 gin and tonic, rum and coke, etc., (50 ml): **2** units
- 1 glass of whisky or brandy (50 ml): **2** units

Assessment of alcohol consumption:

Women ♀	Men ♂	LIV POINTS
NO ALCOHOL CONSUMPTION		10
Up to 7 units/week (*)	Up to 14 units/week (*)	5
8-12 units/week	15-21 units/week	0
Intensive or binge drinking:<br>4 units in one session of 3-4 h	intensive binge drinking:<br>6 **units** in one session of 3-4 h	
13-17 units/week	22-28 units/week	- 5
Over 17 units/week	Over 28 units/week	- 10

(*) Always in non-intensive form (which would score 0 points)

SCORE FOR ALCOHOL CONSUMPTION (Maximum 10 points)

3.2. SMOKING AND OTHER TOBACCO USES

LIV POINTS

Questions	Answers
**Do you currently smoke*?**<br>Answer YES if you do, whether habitually (daily) or occasionally.<br>* Includes the use of cigarettes, cigars, pipes, e-cigarettes/vaping devices, hookahs, etc.<br>**If you do:**<br>- How many cigarettes/e-cigarettes or cigars or hookahs per day?	NO: 15 POINTS<br>YES, occasionally = 0 points<br>YES, 1-10 cigarettes/day (or 1 hookah, or 1 cigar/day) = -5<br>Si, 11-20 cigarettes/day (or 2 hookahs or 2 cigar/day) = - 10<br>Yes, over 20 cigarettes/day (or ≥ 3 hookahs or 3 cigar/day) = - 15 points

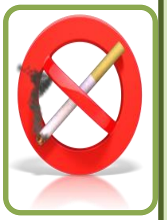

3.3. CONSUMPTION OF OTHER DRUGS

LIV POINTS

Questions	Answers
**1. Do you use any kind of drug*?**<br>*Cannabis/marijuana/hash, cocaine, heroin, ecstasy, synthetic drugs, others…	- No = 0 points<br>- Yes, occasionally = - 5 points<br>- Yes, daily = - 10

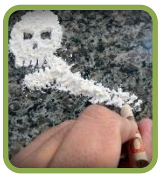

TOTAL SCORE FOR ALL THESE HABITS (Maximum 25 points)

4. EMOTIONAL WELLBEING

	Questions	Answers	LIV POINTS
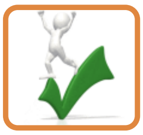	1. I am happy with who I am (at peace with myself)	Always or nearly always: 2 points<br>Sometimes: 1 point<br>Never or hardly ever: 0 points	
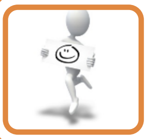	2. I am happy with my life	Always or nearly always: 2 points<br>Sometimes: 1 point<br>Never or hardly ever: 0 points	
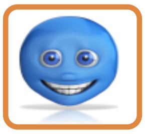	3. I am happy and in good humour	Always or nearly always: 2 points<br>Sometimes: 1 point<br>Never or hardly ever: 0 points	
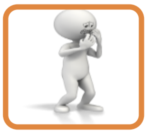	4. I feel stressed, worried or irritable	Always or nearly always: 0 points<br>Sometimes: 1 point<br>Never or hardly ever: 2 points	
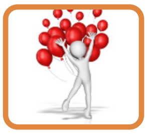	5. I feel motivated to do new activities (leisure/work/sporting)	Always or nearly always: 2 points<br>Sometimes: 1 point<br>Never or hardly ever: 0 points	
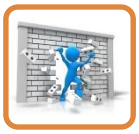	6. I feel I can overcome problems	Always or nearly always: 2 points<br>Sometimes: 1 point<br>Never or hardly ever: 0 points	
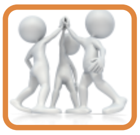	7. I have people with whom I have a good relationship and with whom I can share my worries	Always or nearly always: 2 points<br>Sometimes: 1 point<br>Never or hardly ever: 0 points	
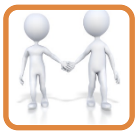	8. I feel loved by those who are important to me	Always or nearly always: 2 points<br>Sometimes: 1 point<br>Never or hardly ever: 0 points	
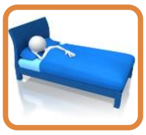	9. I sleep and get up refreshed	Always or nearly always: 2 points<br>Sometimes: 1 point<br>Never or hardly ever: 0 points	
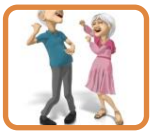	10. I have enough leisure time	Always or nearly always: 2 points<br>Sometimes: 1 point<br>Never or hardly ever: 0 points	

SCORE FOR EMOTIONAL WELLBEING (Maximum 20 points)

5. SAFETY AND NON-INTENTIONAL INJURIES

	Questions	Answers	LIV POINTS
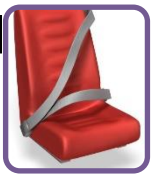	1. Do you wear a seatbelt in a car and/or a helmet when on a motorbike (or when cycling or skating/skate boarding [when indicated]), whether you are driving or a passenger?	YES: 2 points<br>Sometimes: 1 point<br>NO: 0 points	
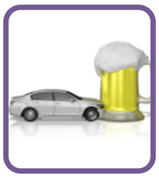	2. Do you follow the recommendation not to drive under the influence of alcohol or drugs, and decline to be driven by those who are?	YES: 2 points<br>Sometimes: 1 point<br>NO: 0 points	
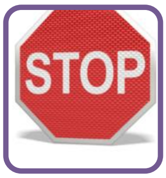	3. Do you follow road regulations, as a driver e.g., keeping to speed limits, not driving and using your telephone in any way, and when a pedestrian do you, e.g., cross roads where it is safe and appropriate?	YES: 2 points<br>Sometimes: 1 point<br>NO: 0 points	
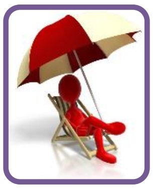	4. Do you follow indications relating to swimming in the sea, in rivers, lakes and swimming pools etc.? E.g., Do you swim where there are lifeguards and obey red flag warnings, avoid swimming at night/after drinking, dive only in inappropriate places	YES: 2 points<br>Sometimes: 1 point<br>NO: 0 points	
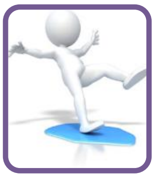	5. Do you follow basic recommendations to prevent domestic accidents (e.g., have regular gas inspections, ventilate rooms to remove heater fumes etc., handle dangerous substances (such as cleaning products) with care, fins ways to prevent falls [especially for the elderly], by using anti-slip patches in the shower or bathtub, by removing mats and ensuring good lighting)	YES: 2 points<br>Sometimes: 1 point<br>NO: 0 points	

SCORE FOR SAFETY AND NON-INTENTIONAL LESIONS (Maximum 10 points)

THE '*PONTE A 100*' HEALTHY LIFESTYLE QUESTIONNAIRE

TOTAL LIV POINTS

FOOD HABITS

PHYSICAL ACTIVITY

SMOKING AND USE OF ALCOHOL OTHER DRUGS

EMOTIONAL WELLBEING

SAFETY AND NON-INTENTIONAL INJURIES

YOUR LIFESTYLE INDEX VALUE

- ✓ The **AIM** is to score the **MAXIMUM 100 LIV points**.
  The higher your Lifestyle Index Value, the more healthy your lifestyle is.
- ✓ **PLAN CHANGES** for the items that scored below-maximum LIV points.
- ✓ **YOU CAN CHANGE YOUR LIFESTYLE AND MAKE IT MORE HEALTHY!**

UNIVERSITAS COMPLUTENSIS MATRITENSIS

Comunidad de Madrid

CONSEJERÍA DE SANIDAD
