# Supplementary material for: Design and Content Validation using Expert Opinions of an Instrument Assessing the Lifestyle of Adults: The ‘PONTE A 100’ Questionnaire
Source: Healthcare (Basel). 2023 Jul 16;11(14):2038. doi: 10.3390/healthcare11142038 (PMC10379326; doi:10.3390/healthcare11142038)
Supplement: Supplementary file 1 [file healthcare-11-02038-s001.zip › Supplementary File S1_Questionnaire in Spanish.pdf]

CONSEJERÍA DE SANIDAD

# CUESTIONARIO DE ESTILO DE VIDA SALUDABLE: “PONTE A 100”

Los **estilos de vida saludable** hacen referencia a un conjunto de comportamientos, hábitos y costumbres cotidianas que llevan a cabo las personas para mantener, de manera adecuada, su cuerpo y su mente a lo largo de todo el ciclo vital. Contribuyen a mejorar el bienestar personal y la calidad de vida, a mantenerse sano y a disminuir la discapacidad y el dolor.

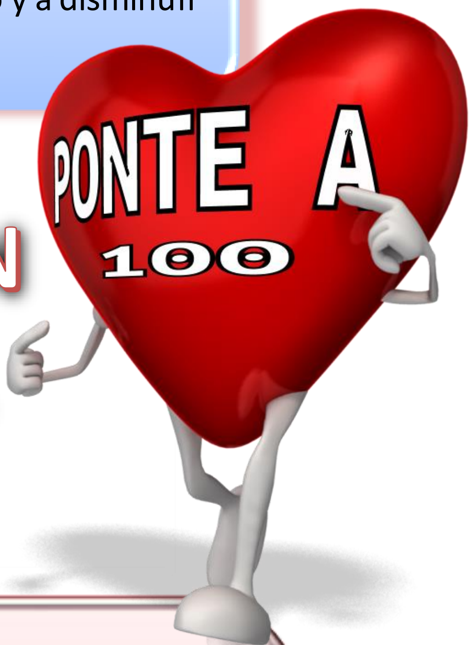


## ¿TIENE USTED UN ESTILO DE VIDA SALUDABLE?

- ✓ **Cumplimente** el siguiente cuestionario y conozca si su estilo de vida es saludable.
- ✓ Consta de **33 preguntas agrupadas en cinco apartados:** ‘Alimentación’, ‘Actividad física y sedentarismo’, ‘Hábitos tóxicos (consumo de alcohol, tabaco y otras drogas)’, ‘Bienestar emocional’ y ‘Seguridad y Lesiones no intencionales’.
- ✓ **Valore** cada una de las **preguntas y asigne los puntos** de la opción que más se adapte a su situación personal en los **últimos 3 meses.** No hay respuestas correctas e incorrectas.
- ✓ Obtenga la puntuación para cada uno de los **apartados y la puntuación final** sumando la obtenida para cada uno de los apartados con un máximo de **100 puntos.**

# 1.- ALIMENTACION

## PREGUNTAS DE VALORACIÓN — RESPUESTAS — PUNTOS

| | PREGUNTAS DE VALORACIÓN | RESPUESTAS | PUNTOS |
|---|---|---|---|
| 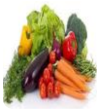 | **1. ¿Cuántas raciones de verduras u hortalizas (no se incluyen patatas) consume al día? (1 ración*: 150-200g)**<br>* Plato grande de ensalada variada, de verdura cocida, de puré de verduras o de gazpacho/salmorejo; 1 tomate grande; 2 zanahorias. Guarnición=1/2 ración. | Más de 1 = 2 puntos<br>1 ración = 1 punto<br>Ninguna = 0 puntos | |
| 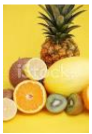 | **2. ¿Cuántas raciones de fruta consume al día?**<br>**(1 ración*: 150-200 g)**<br>* 1 pieza mediana (naranja/manzana/plátano…), 2 piezas pequeñas (ciruelas/mandarinas/albaricoques…), 1 taza/bol de cerezas/fresas/uvas, 1 raja/rodaja de sandía/melón/piña | Más de 2= 2 puntos<br>1-2 piezas = 1 punto<br>Ninguna = 0 puntos | |
| 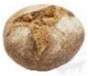 | **3.- ¿Cuantas raciones* de cereales integrales (pan, pasta, arroz…) consume al día?**<br>*1 ración de pan integral:40-60 g (2-3 rebanadas);<br>1 ración de arroz/ pasta integral:60-80 g (3 cucharadas en crudo o 1 plato mediano cocinado) | Más de 2 = 3 puntos<br>2= 2 puntos<br>1 = 1 punto<br>Ninguna = 0 puntos | |
| 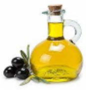 | **4. ¿Consume al menos 3 cucharadas soperas (30 ml) de aceite al día (crudo, frito…)?** | - Si, de Aceite de Oliva Virgen (o virgen extra) = 2 puntos<br>- Si, de aceite de Oliva = 1 punto<br>- No consume esa cantidad de aceite o consume otro tipo de aceite/margarina/mantequilla = 0 puntos | |
| 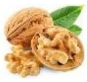 | **5. ¿Cuántas raciones* de frutos secos (nueces, avellanas, almendras, anacardos, pistachos) consume a la semana?**<br>*1 ración: 1 puñado de 20-30 g | Crudos (al natural): De 4 a 7 raciones/semana = 2 puntos<br>Crudos o Tostados (1-3 raciones) = 1 punto<br>No consumo esas cantidades o los consumo fritos, salados o dulces = 0 puntos | |
| 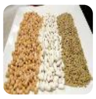 | **6. ¿Cuántas raciones* de legumbres (garbanzos, lentejas, alubias…) consume a la semana?**<br>*1 ración: 60-80 g crudo (1 puñado/taza), 150-200 g cocido (1 plato hondo mediano o 2-3 cazos de servir con caldo) | Más de 2 = 2 puntos<br>1 - 2 = 1 punto<br>Ninguna = 0 puntos | |
| 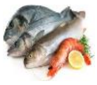 | **7. ¿Cuántas raciones* de pescado/marisco consume a la semana?**<br>*1 ración=100-150g (1 filete/rodaja mediana, 3-4 piezas de marisco) | Más de 2 = 2 puntos<br>1 - 2 = 1 punto<br>Ninguna = 0 puntos | |
| 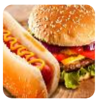 | **8. ¿Cuántas raciones de carnes rojas* (vacuno, cordero, cerdo...) o carnes procesadas** (hamburguesas, salchichas, embutidos, nuggets de pollo…) consume a la semana?**<br>*1 ración=100-150g: filete mediano<br>**1 ración: 3-4 lonchas de embutido;1 hamburguesa; 3 albóndigas. | Menos de 3 = 3 puntos<br>3 - 4 = 1 punto<br>Más de 4 = 0 puntos | |
| 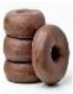<br>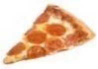 | **9. ¿Consume algún producto altamente procesado o ultraprocesado(*) a la semana?**<br>(*) Snacks dulces y salados, chucherías, pan envasado, pan de molde, bizcochos, bollos, pasteles, galletas, cereales de desayuno refinados/azucarados, palitos de pescado, salsas comerciales, pizza, batidos, helados, sopas 'instantáneas', pastillas de caldo… y otros alimentos precocinados/listos para consumir con exceso de sal, azúcar, aceites refinados o conservantes/ aditivos. (Se excluyen alimentos poco procesados como las frutas y verduras envasadas, los encurtidos o las legumbres cocidas en bote) | Menos de 3 = 3 puntos<br>3 - 4 = 1 punto<br>Mas de 4 = 0 puntos | |
| 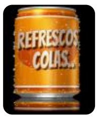 | **10. ¿Consume alguna bebida azucarada/edulcorada (refrescos, bebidas energéticas, batidos, zumos envasados…) a diario/casi a diario?** | No = 2 puntos<br>Si, bebidas edulcoradas ('light', 'zero', 'sin azúcar') = 1 punto<br>Sí, bebidas azucaradas= 0 puntos | |
| 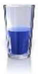 | **11. ¿Bebe agua a lo largo del día y/o con las comidas?** | Sí, 4 o más vasos*= 2 puntos<br>Sí, menos de 4 vasos*= 1 punto.<br>No= 0 puntos<br>* 1 vaso= 200ml | |

**PUNTUACIÓN TOTAL ALIMENTACIÓN (Máximo 25 puntos)**

# 2.- ACTIVIDAD FÍSICA Y SEDENTARISMO

| | PREGUNTAS DE VALORACIÓN | RESPUESTAS | PUNTOS |
|---|---|---|---|
| 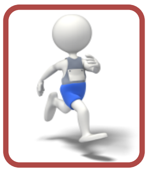 | **1.** ¿Cuántas veces por semana realiza usted, al menos, 25 minutos de **actividad física INTENSA**? | 3 o más = 12 puntos<br>1-2 = 6 puntos<br>Nunca = 0 puntos | 1 |
| 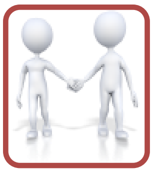 | **2.** ¿Cuántas veces por semana realiza usted, al menos, 30 minutos de **actividad física MODERADA**? | 5 o más = 12 puntos<br>3-4 = 6 puntos<br>1-2 = 3 puntos<br>Nunca = 0 puntos | 2 |
| 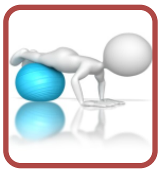 | **3**. ¿Cuántas veces por semana realiza **ejercicios de fortalecimiento muscular***?<br>* Pesas/Mancuernas, máquinas de pesas, bandas de resistencia o actividades con el propio peso corporal, (flexiones en el suelo o contra la pared, sentadillas, abdominales, …) | 2 o más = 4 puntos<br>1 = 2 puntos<br>Nunca = 0 puntos | 3 |
| 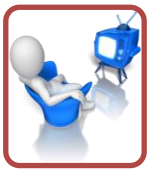 | **4**. A lo largo del día, durante el tiempo que se encuentra sentado/a, reclinado/a o tumbado/a, ¿realiza **descansos activos cada 60-90 minutos** dando un breve paseo o con sesiones cortas de estiramientos? | Siempre o casi siempre: 4 puntos<br>Algunas veces: 2 puntos<br>Nunca o Casi Nunca: 0 [illegible] | 4 |

Los tiempos de actividad física también se pueden alcanzar sumando periodos más cortos de actividad, a partir de 10 minutos.

**NOTA: LAS PREGUNTAS 1 Y 2 SON COMPLEMENTARIAS, PERMITIENDO DIFERENTES COMBINACIONES DE ACTIVIDAD, PERO SIEMPRE POR UN MÁXIMO DE 12 PUNTOS.**

Puntuación 1+ 2: **(Máximo 12 puntos)** + Puntuación 3: **(Máximo 4 puntos)** + Puntuación 4: **(Máximo 4 puntos)** = **PUNTUACIÓN TOTAL ACTIVIDAD FÍSICA (Máximo 20 puntos)**

La **intensidad** es el esfuerzo que tenemos que hacer para llevar a cabo una actividad física

La intensidad de diferentes formas de actividad física varía de una persona a otra y depende de lo ejercitado que esté cada uno y de su forma física. Por ello, los ejemplos que se exponen a continuación son orientativos y pueden variar de una persona a otra

**1.- INTENSA o VIGOROSA (Requiere una gran cantidad de esfuerzo y provoca una respiración rápida y un aumento sustancial de la frecuencia cardíaca)**: footing, ascender a paso rápido o trepar por una ladera, desplazamientos rápidos en bicicleta, aerobic, natación rápida, deportes y juegos competitivos (fútbol, voleibol, hockey, baloncesto, patinaje, squash…)

**2.- MODERADA (Requiere un esfuerzo moderado, que acelera de forma perceptible el ritmo cardiaco y el respiratorio, pero aún se puede hablar sin sentir que falta el aire)**: caminar a paso rápido (7000-8000 pasos/día), bailar, participación activa en juegos y deportes con niños, pasear en bicicleta, nadar por placer…

# 3.- CONSUMO DE ALCOHOL, TABACO Y OTRAS DROGAS

## 3.1.- CONSUMO DE ALCOHOL

1.- Habitualmente, ¿cuántas **unidades** de bebida alcohólica suele consumir en una **semana**?

**Nº unidades**

### UNIDADES ALCOHÓLICAS DE CADA BEBIDA

- 1 copa de vino tinto, blanco o cava (100 ml): **1** unidad
- 1 caña de cerveza o 1 vaso de sidra (200 ml): **1** unidad
- 1 carajillo (25 ml) o 1 vermut (50 ml): **1** unidad
- 1 chupito de anís, pacharán, licor (30 ml): **1** unidad
- 1 tercio de cerveza (330 ml): **1,5** unidades
- 1 combinado de gin tonic, ron cola... (50 ml): **2** unidades
- 1 copa de whisky o coñac (50 ml): **2** unidades

### VALORACIÓN DEL CONSUMO DE ALCOHOL:

| Mujeres ♀ | Hombres ♂ | PUNTOS |
|---|---|---|
| NINGÚN CONSUMO DE ALCOHOL (CONSUMO CERO) | | 10 |
| Consumo de hasta 7 unidades/semana (*) | Consumo de hasta 14 unidades/semana (*) | 5 |
| Consumo entre 8 y 12 unidades/semana | Consumo entre 15 y 21 unidades/semana | 0 |
| Consumo intensivo o 'binge drinking': 4 unidades en una sesión de 3-4 h | Consumo intensivo o 'binge drinking': 6 unidades en una sesión de 3-4 h | |
| Consumo entre 13 y 17 unidades/semana | Consumo entre 22 y 28 unidades/semana | - 5 |
| Consumo superior a 17 unidades/semana | Consumo superior a 28 unidades/semana | - 10 |

(*) Siempre que este consumo no se realice en forma de consumo intensivo, en cuyo caso la puntuación sería 0

**PUNTUACIÓN TOTAL CONSUMO DE ALCOHOL (Máximo 10 puntos)**

# 3.2.- CONSUMO DE TABACO

| PREGUNTAS DE VALORACIÓN | RESPUESTAS | PUNTOS |
|---|---|---|
| 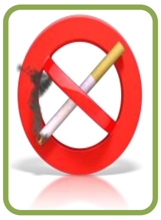 **1.- ¿Fuma en la actualidad?:**<br>Responda SI en el caso de que fume (*) en la actualidad, bien de forma habitual (diariamente) o de forma ocasional.<br>* Cigarros, puros, pipas, sistemas electrónicos de administración de nicotina/cigarrillos electrónicos/vapeadores, pipas de agua/cachimba/shisha/narguiles, …<br>**En caso de fumadores, señalar:**<br>- Nº de cigarrillos/cigarrillos electrónicos o cachimbas o puros/día: | NO: 15 PUNTOS<br>SI, ocasionalmente = 0 puntos<br>Sí, 1-10 cig/día (o 1 cachimba o 1puro/día) = -5<br>Si, 11-20 cig/día (o 2 cachimbas o 2 puros/día) = - 10<br>Sí, más de 20 cig/día (o ≥ 3 cachimbas o 3 puros /día) = - 15 puntos | |

# 3.3.- CONSUMO DE OTRAS DROGAS

| PREGUNTAS DE VALORACIÓN | RESPUESTAS | PUNTOS |
|---|---|---|
| 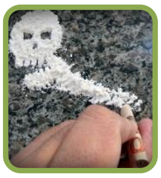 **1.-¿Consume algún otro tipo de droga*?.**<br>* Cannabis/marihuana/hachís, cocaína, heroína, éxtasis, drogas de síntesis, otro tipo de drogas… | - No = 0 puntos<br>- Sí, ocasionalmente = - 5 puntos<br>- Sí, a diario = - 10 | |

**PUNTUACIÓN TOTAL HÁBITOS TÓXICOS (Máximo 25 puntos)**

# 4.- BIENESTAR EMOCIONAL

| | PREGUNTAS DE VALORACIÓN | RESPUESTAS | PUNTOS |
|---|---|---|---|
| 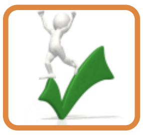 | 1.- Me siento satisfecho/a de quién/como soy (estoy a gusto conmigo mismo/a) | Siempre o casi siempre: 2 puntos<br>Algunas veces: 1 punto<br>Nunca o Casi Nunca: 0 puntos | |
| 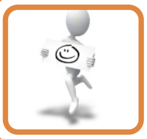 | 2.- Estoy contento/a con la vida que llevo | Siempre o casi siempre: 2 puntos<br>Algunas veces: 1 punto<br>Nunca o Casi Nunca: 0 puntos | |
| 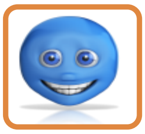 | 3.- Estoy alegre y de buen humor | Siempre o casi siempre: 2 puntos<br>Algunas veces: 1 punto<br>Nunca o Casi Nunca: 0 puntos | |
| 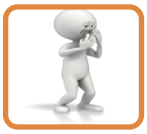 | 4.- Me siento estresado/a, nervioso/a, angustiado/a, irritable… | Siempre o casi siempre: 0 puntos<br>Algunas veces: 1 punto<br>Nunca o Casi Nunca: 2 puntos | |
| 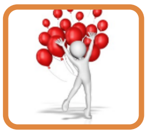 | 5.- Me siento motivado/a a la hora de realizar nuevas actividades (ocio/ laborales/ deportivas) | Siempre o casi siempre: 2 puntos<br>Algunas veces: 1 punto<br>Nunca o Casi Nunca: 0 puntos | |
| 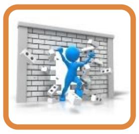 | 6.-Me siento capaz de superar las dificultades que se me presentan | Siempre o casi siempre: 2 puntos<br>Algunas veces: 1 punto<br>Nunca o Casi Nunca: 0 puntos | |
| 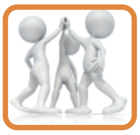 | 7.- Tengo personas en mi entorno con las que me relaciono satisfactoriamente | Siempre o casi siempre: 2 puntos<br>Algunas veces: 1 punto<br>Nunca o Casi Nunca: 0 puntos | |
| 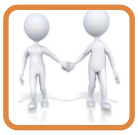 | 8.- Me siento querido/a por las personas importantes para mi | Siempre o casi siempre: 2 puntos<br>Algunas veces: 1 punto<br>Nunca o Casi Nunca: 0 puntos | |
| 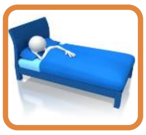 | 9.- Duermo bien, me levanto descansado/a. | Siempre o casi siempre: 2 puntos<br>Algunas veces: 1 punto<br>Nunca o Casi Nunca: 0 puntos | |
| 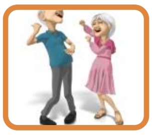 | 10.- Tengo suficiente tiempo de ocio. | Siempre o casi siempre: 2 puntos<br>Algunas veces: 1 punto<br>Nunca o Casi Nunca: 0 puntos | |

**PUNTUACIÓN TOTAL BIENESTAR EMOCIONAL (Máximo 20 puntos)**

# 5.- SEGURIDAD Y LESIONES NO INTENCIONALES

| | PREGUNTAS DE VALORACIÓN | RESPUESTAS | PUNTOS |
|---|---|---|---|
| 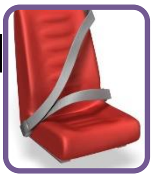 | 1.- Llevo el cinturón de seguridad cuando viajo en coche y/o el casco si voy en moto (o en bicicleta, patines o patinete, cuando esté indicado), tanto como conductor como acompañante. | SI: 2 puntos<br>A veces: 1 punto<br>NO: 0 puntos | |
| 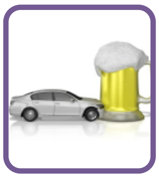 | 2.- Sigo las recomendaciones de no conducir bajo la influencia del alcohol u otras drogas, ni viajo con alguien que conduzca bajo su influencia. | SI: 2 puntos<br>A veces: 1 punto<br>NO: 0 puntos | |
| 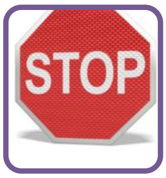  | 3.- Respeto las normas de tráfico como conductor/a y/o peatón/a: límites de velocidad, cruzar por sitios autorizados, evitar distracciones con el teléfono móvil (enviar WhatsApp®/redes sociales/llamadas)… | SI: 2 puntos<br>A veces: 1 punto<br>NO: 0 puntos | |
| 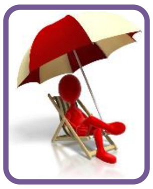 | 4. Respeto las normas de seguridad(*) relacionadas con el baño en playas, ríos, pantanos, embalses y piscinas.<br>(*) Bañarse en playas con vigilancia; no bañarse si esta la bandera roja, si es de noche o se ha consumido alcohol; no tirarse de cabeza en zonas desconocidas o en las que está prohibido; utilizar protección solar | SI: 2 puntos<br>A veces: 1 punto<br>NO: 0 puntos | |
| 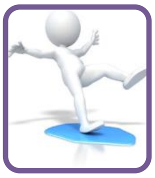 | 5.- Sigo las recomendaciones básicas (*) para evitar los accidentes en el hogar<br>(*) Revisión periódica de instalaciones de gas, ventilar a diario salas con estufas o braseros, manejo con prudencia de sustancias peligrosas como los productos de limpieza, prevención de caídas en personas mayores (instalación de tiras antideslizantes en duchas y bañeras, evitar alfombras, buena iluminación…) | SI: 2 puntos<br>A veces: 1 punto<br>NO: 0 puntos | |

**PUNTUACIÓN TOTAL SEGURIDAD Y LESIONES NO INTENCIONALES (Máximo 10 puntos)**

# CUESTIONARIO DE ESTILO DE VIDA SALUDABLE EN ATENCIÓN PRIMARIA: "PONTE A 100"

## PUNTUACIÓN TOTAL

| | |
|---|---|
| ALIMENTACION | |
| ACTIVIDAD FÍSICA | |
| CONSUMO DE ALCOHOL, TABACO Y OTRAS DROGAS | |
| BIENESTAR EMOCIONAL | |
| SEGURIDAD Y LESIONES NO INTENCIONALES | |

## PUNTUACIÓN TOTAL (ISEV*)

- ✓ El **OBJETIVO** es **ALCANZAR LA MAXIMA PUNTUACIÓN** (100 puntos). Cuanto mayor es la puntuación, más saludable se considera su estilo de vida.
- ✓ **REVISE** los **APARTADOS** donde haya alcanzado **MENOR PUNTUACIÓN** y planifique por dónde quiere empezar a cambiar.
- ✓ Siempre es **POSIBLE CAMBIAR EL ESTILO DE VIDA** para hacerlo más **SALUDABLE.**

*** ISEV: ÍNDICE SINTÉTICO DE ESTILOS DE VIDA**

Comunidad de Madrid | CONSEJERÍA DE SANIDAD
